# Surveillance of genetic markers associated with *Plasmodium falciparum* resistance to artemisinin-based combination therapy in Pakistan, 2018–2019

**DOI:** 10.1186/s12936-020-03276-8

**Published:** 2020-06-08

**Authors:** Abdul Qader Khan, Leyre Pernaute-Lau, Aamer Ali Khattak, Sanna Luijcx, Berit Aydin-Schmidt, Mubashir Hussain, Taj Ali Khan, Farees Uddin Mufti, Ulrika Morris

**Affiliations:** 1 Department of Microbiology, Kohat University of Science and Technology, Kohat, Pakistan; 2 Department of Microbiology, Tumor and Cell Biology, Karolinska Institutet, Stockholm, Sweden; 3 Biosystems and Integrative Science Institute, Faculty of Sciences of the University of Lisbon, Lisbon, Portugal; 4 Department of Medical Lab Technology, The University of Haripur, Haripur, Pakistan; 5 Department of Biotechnology, Quaid-i-Azam University, Islamabad, Pakistan

**Keywords:** *Plasmodium falciparum*, Pakistan, Artemisinin-based combination therapy, Artemether–lumefantrine, Chloroquine, Drug resistance, *P. falciparum coronin*, *P. falciparum kelch 13* propeller domain, *pfcrt*, *pfmdr1*

## Abstract

**Background:**

The spread of artemisinin resistance in the Greater Mekong Subregion of Southeast Asia poses a significant threat for current anti-malarial treatment guidelines globally. The aim of this study was to assess the current prevalence of molecular markers of drug resistance in *Plasmodium falciparum* in the four provinces with the highest malaria burden in Pakistan, after introducing artemether–lumefantrine as first-line treatment in 2017.

**Methods:**

Samples were collected during routine malaria surveillance in Punjab, Sindh, Baluchistan, and Khyber Pakhtunkhwa provinces of Pakistan between January 2018 and February 2019. *Plasmodium falciparum* infections were confirmed by rapid diagnostic test or microscopy. *Plasmodium falciparum* positive isolates (n = 179) were screened by Sanger sequencing for single nucleotide polymorphisms (SNPs) in the *P. falciparum kelch 13* (*pfk13)* propeller domain and in *P. falciparum coronin* (*pfcoronin*). SNPs in *P. falciparum multidrug resistance 1 (pfmdr1)* N86Y, Y184F, D1246Y and *P. falciparum chloroquine resistance transporter* (*pfcrt)* K76T were genotyped by PCR-restriction fragment length polymorphism.

**Results:**

No artemisinin resistance associated SNPs were identified in the *pfk13* propeller domain or in *pfcoronin*. The *pfmdr1* N86, 184F, D1246 and *pfcrt* K76 alleles associated with reduced lumefantrine sensitivity were present in 83.8% (150/179), 16.9% (29/172), 100.0% (173/173), and 8.4% (15/179) of all infections, respectively. The chloroquine resistance associated *pfcrt* 76T allele was present in 98.3% (176/179) of infections.

**Conclusion:**

This study provides an update on the current prevalence of molecular markers associated with reduced *P. falciparum* sensitivity to artemether and/or lumefantrine in Pakistan, including a first baseline assessment of polymorphisms in *pfcoronin*. No mutations associated with artemisinin resistance were observed in *pfk13* or *pfcoronin*. However, the prevalence of the *pfmdr1* N86 and D1246 alleles, that have been associated with decreased susceptibility to lumefantrine, remain high. Although clinical and molecular data suggest that the current malaria treatment guidelines for *P. falciparum* are presently effective in Pakistan, close monitoring for artemisinin and lumefantrine resistance will be critical to ensure early detection and enhanced containment of emerging ACT resistance spreading across from Southeast Asia.

## Background

Despite ongoing global efforts to control and eradicate malaria, the disease remains a significant contributor to morbidity and mortality worldwide. According to the World Health Organization (WHO), there were over 228 million cases of malaria in 2018, resulting in approximately 405,000 deaths [1]. In Pakistan, malaria transmission is moderate, with approximately 60% of the population (123 million people) at high risk of contracting malaria. According to the Pakistan malaria annual report there were more than 300,000 confirmed malaria cases reported in 2018 [2]. Among these cases, 84% were caused by *Plasmodium vivax*, 15% by *Plasmodium falciparum*, and 1% were mixed *P. falciparum* and *P. vivax* infections.

Malaria control relies on a handful of interventions including prompt and effective treatment with artemisinin-based combination therapy (ACT); a strategy currently threatened by *P. falciparum* resistance to artemisinin and its partner drugs spreading across Southeast Asia [3, 4]. Artemisinin resistance was first reported in Cambodia in 2009 [5, 6], and is now widespread across the Greater Mekong Subregion [4, 7, 8]. It is phenotypically characterized by delayed parasite clearance following treatment, which has been associated with a number of single nucleotide polymorphisms (SNPs), including F446I, Y493H, R539T, I543T, and C580Y, in the propeller domain of the *P. falciparum kelch 13* (*pfk13*) gene [4, 9, 10]. Possible independent emergence of the *pfk13* C580Y mutation has been reported in Guyana [11] and Papua New Guinea [12], and candidate *pfk13* resistance mutations (P574L and A675V), which are common in southeast Asia and have been associated with delayed parasite clearance, have been identified in Rwanda [13]. Concomitant emergence of tolerance and/or resistance to the long-acting partner drugs in artemisinin-based combinations (namely, piperaquine, mefloquine, sulfadoxine-pyrimethamine, lumefantrine, and amodiaquine), has resulted in ACT failures in several areas of southeast Asia [3, 14–16]. Recently, delayed parasite clearance associated with polymorphisms in *pfk13* has been reported in Eastern India after treatment with artesunate-sulfadoxine-pyrimethamine (AS + SP) [17, 18].

The availability of molecular markers for both artemisinin- and partner drug resistance has greatly aided close surveillance of the emergence and spread of ACT resistance. In addition to *pfk13,* SNPs in the *P. falciparum* actin-binding protein coronin (*pfcoronin*) gene (G50E, R100K, and E107V) have recently been identified to confer reduced in vitro sensitivity to artemisinin [19]. Whilst these mutations have yet to be found in vivo in samples from Africa or elsewhere [20], it has been suggested that *pfcoronin* mutants could emerge as a non-*pfk13* type of artemisinin resistance in natural settings [19]. Moreover, polymorphisms in the *P. falciparum* multidrug resistance 1 (*pfmdr1*) and *P. falciparum* chloroquine resistance transporter (*pfcrt*) genes have been associated with decreased susceptibility to chloroquine, and important ACT partner drugs including lumefantrine, mefloquine, and amodiaquine [21–27].

After establishment of chloroquine resistance in Pakistan, the WHO recommended AS + SP for treatment of uncomplicated *P. falciparum* infections in 2007. However, after rapid increases in AS + SP treatment failure rates in Somalia and Sudan (ranging from 12% to 22%) [1, 4], and the recent reports from India [17, 18], the national treatment guidelines were updated in 2017 with artemether–lumefantrine (AL) given together with a single low dose of primaquine as recommended first-line treatment for uncomplicated *P. falciparum* malaria [1].

This study aims to provide an update on the current prevalence of molecular markers associated with drug resistance after introduction of AL as first-line treatment, in the four provinces with the highest malaria burden in Pakistan. This includes a first baseline assessment of polymorphisms in the *pfcoronin* gene and prevalence of SNPs in the *pfk13* propeller region, that have been associated with reduced sensitivity to artemisinin, as well as the proportions of infections harbouring the *pfmdr1* N86, 184F, D1246 and *pfcrt* K76 alleles that have been associated with reduced sensitivity to lumefantrine [25, 28–36].

## Methods

### Study sites

**Fig. 1:**
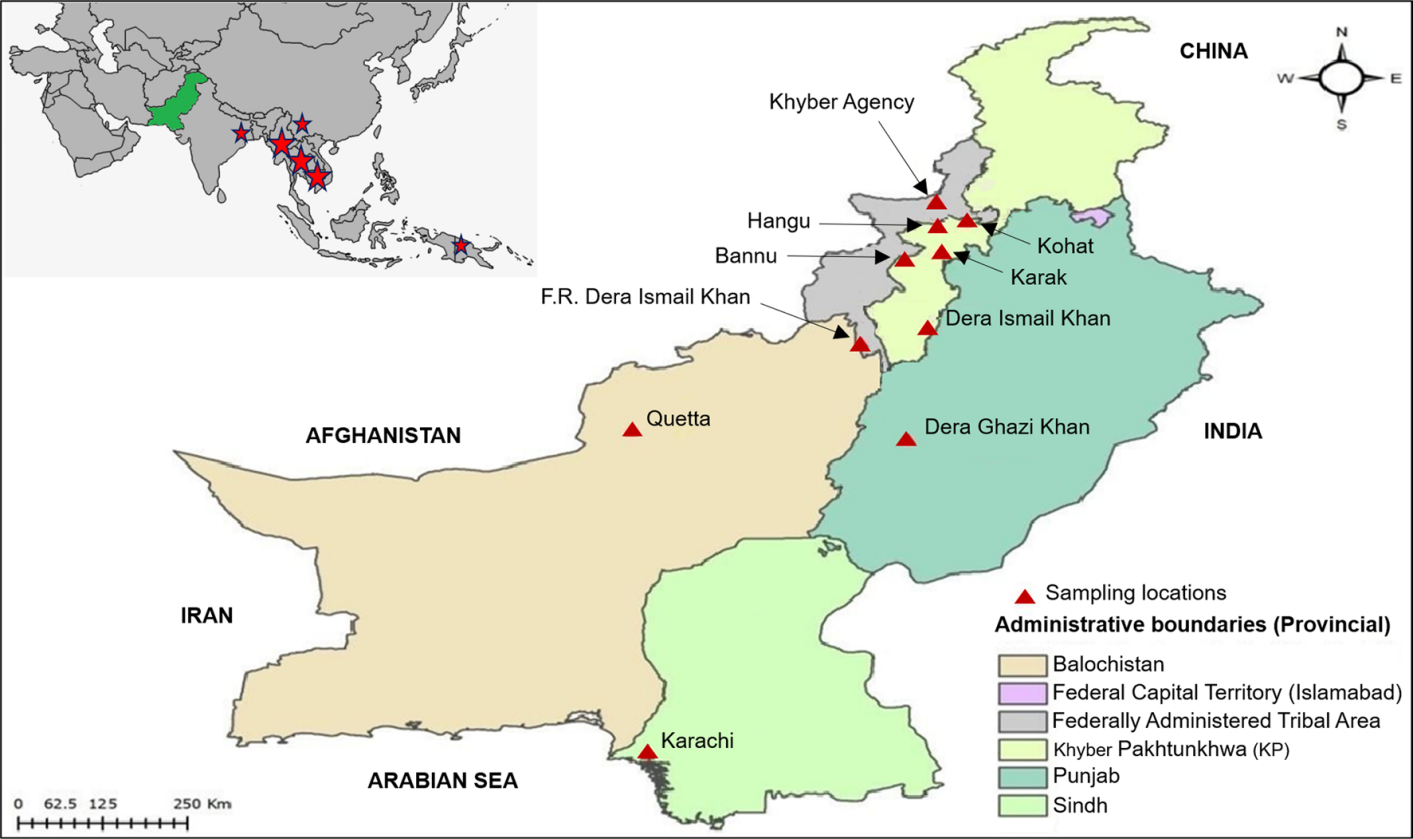
Map of Pakistan showing the geographical location of the 10 different sample collection sites. Pakistan is highlighted in green in the top left panel; red stars identify locations of reported Artemisinin resistance. In the enlarged map of Pakistan, red triangles indicate the 10 different sample collection sites in four provinces

Seventeen government and private hospitals in the four provinces with the highest malaria burden in Pakistan (Khyber Pakhtunkhwa (KPK), Sindh, Balochistan, and Punjab) were invited to provide blood samples during routine malaria surveillance. Ten facilities responded and were included in this study: Bannu, Kohat, Karak, Dera Ismail Khan, Frontier Region (FR) Khyber, FR Dera Ismaeel (DI) Khan, and Hangu in KPK province; Karachi in Sindh Province; Quetta in Balochistan Province; and Dera Ghazi Khan in Punjab Province (Fig. 1). Symptomatic, febrile patients who visited government and/or private hospitals in these selected sites between January 2018 and February 2019 were registered in this study. Written informed consent was obtained from all participants/guardians before collecting clinical history and venous blood samples.

### Samples storage and DNA extraction

Approximately 5 ml of whole blood was collected in EDTA tubes from 209 patients with malaria infections confirmed by rapid diagnostic test and/or microscopy. Samples were shipped to Karolinska Institutet, Stockholm, Sweden for subsequent molecular analysis. DNA was extracted from 200 μl of whole blood using the QIAamp DNA mini kit (QIAGEN, Inc., Germany), following the manufacturer’s guidelines. Extracted DNA was stored at − 20 °C.

### Sequencing of the *pfk13* propeller domain and *pfcoronin*

The 849 base pair fragment covering amino acid positions 407–689 in the *pfk13*-propeller domain was amplified with Q5 high-fidelity polymerase (New England Biolabs, UK) using a previously described nested polymerase chain reaction (PCR) protocol [9]. A *P. falciparum* 3D7 positive control and a no template control was included in each PCR. Amplified PCR products were separated by electrophoresis in 1.5% agarose gels stained with GelRed^®^ (Biotium, USA). The 849-base pair fragment was extracted from the gel and purified using GeneJET Gel Extraction Kit (Thermo Fisher Scientific, Lithuania). The purified PCR products from clinical isolates and 3D7 positive control were sent for bidirectional Sanger sequencing at Macrogen, Netherlands. The sequencing data was analysed using Sequencher version 5.1 software (Gene Codes Corporation, USA). Generated sequences were aligned with the reference sequence of 3D7 (PlasmoDB gene ID: PF3D7_1343700). Nested PCR and sequencing were repeated in case of poor-quality sequencing output.

A 368 base pair fragment covering the region in the N-terminal WD40 domain of *pfcoronin* where all three SNPs associated with artemisinin resistance were reported [19], was amplified in a nested PCR. The following primers were used for the primary PCR: Pfcoronin-1st_fwd 5′-TGATTTGTTCATATTATAGGTAC-3′ and Pfcoronin-1st_rev 5′-TATTCTGACAAGTTCCACTTAATA-3′, and for the nested PCR: Pfcoronin-nest_fwd 5′-CATATTATAGGTACCATGGCAAGTT-3′ and Pfcoronin-nest_rev 5′-AGGCTTTCTTCTCATTTTCTATATC-3′. For the primary PCR, 1 µl of DNA was amplified with 1 µM of each primer, 0.2 mM dNTP, 3.75 mM MgCl_2_, 1 × GoTaq reaction buffer, and 0.75U GoTaq^®^ DNA polymerase (Promega, USA), using the following cycling program: 30 s at 90 °C; followed by 30 cycles of 20 s at 90 °C, 30 s at 45 °C, 1.30 min at 68 °C. For the nested PCR, 1 µL of primary PCR products were amplified under the same conditions using the following cycling program: 30 s at 90 °C; followed by 45 cycles of 15 s at 94 °C, 30 s at 50 °C, 1 min at 68 °C. A 3D7 positive control and a no template control was included in each PCR. The nested PCR products were sent for direct bidirectional Sanger sequencing at Macrogen and analysed as described above with 3D7 as a reference (PlasmoDB gene ID: PF3D7_1251200).

### Genotyping of SNPs in *pfmdr1* and *pfcrt*

SNPs at positions *pfmdr1* N86Y, Y184F, and D1246Y and *pfcrt* K76T were genotyped following nested PCR-restriction fragment length polymorphism (RFLP) protocols as summarized in [37]. Laboratory clones 3D7, Dd2, and 7G8 were included as positive and negative restriction controls, and a no template control was included in each PCR. 5 µL of each PCR amplicon was digested overnight with either ApoI, MluCI or EcoV (New England Biolabs, UK), following manufacturer’s instructions. RFLP products were run on 2–2.5% agarose gels stained with GelRed^®^ (Biotium, USA) and documented with a GelDoc™ system.

### Data analysis

The prevalence of each SNP was defined as the proportion of isolates containing the allele of interest, including mixed infections. Isolates containing mixed SNP results at more than one position were excluded from the *pfmdr1* haplotype analysis; frequencies for haplotypes were otherwise calculated as for the SNPs.

## Results

### Study population

In total, 209 uncomplicated *P*. *falciparum* cases from 10 different sites across the country were enrolled in this study between January 2018 and February 2019 (Table 1). Among rapid diagnostic rest and/or microscopy positive malarial isolates, 179 samples were confirmed as *P. falciparum* infections by PCR. Among the 179 PCR confirmed patients, 120 (67%) were male and 59 (33%) were female, with ages ranging from two to 65 years, and a median age of 25 years.

**Table 1:** *Plasmodium falciparum* samples collected from 10 sites in Pakistan, 2018–2019

Province	City	PCR-confirmed *P. falciparum* infection (N)	Sex (%male %female)	Median age (years [IQR^a^])
Khyber Pakhtunkhwa	Karak	10	60/40	35 [10–45]
	Kohat	8	50/50	22 [14–28]
	Dera Ismeel Khan	10	90/10	25 [16–30]
	Bannu	13	85/15	25 [7–36]
	Hangu	5	40/60	25 [20–35]
	FR Khyber	10	80/20	8 [4–20]
	FR DI Khan	12	83/17	13 [9–29]
Punjab	Dera Ghazi Khan	53	60/40	30 [15–40]
Sindh	Karachi	41	60/40	25 [15–37]
Baluchistan	Quetta	17	76/24	28 [21–40]
Total		179	68/32	25 [2–60]

^a^ IQR, interquartile range

### Prevalence of SNPs in *pfk13* and *pfcoronin*

PCR success rates for the nested PCR amplifying the *pfk13* propeller domain and *pfcoronin* were 97.7% (175/179) and 100% (179/179), respectively, of which 121 (69.1%) and 149 (83.2%) generated good quality sequences. No artemisinin resistance associated SNPs were identified in either of the analysed fragments, hence all successfully sequenced samples showed wild-type genotypes in both *pfk13* and *pfcoronin*.

### Prevalence of SNPs in *pfcrt* and *pfmdr1*

**Fig. 2:**
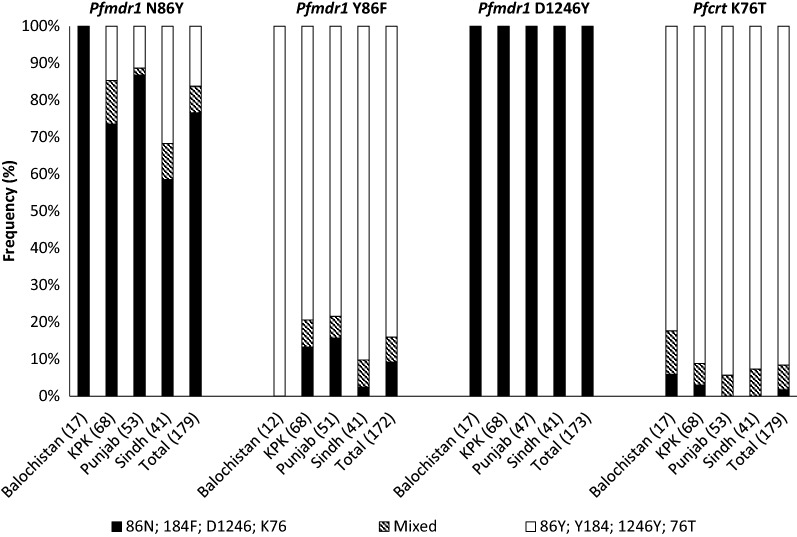
Distribution of molecular markers of drug resistance across four provinces of Pakistan, 2018–2019. Clustered, stacked bar chart showing the prevalence of single nucleotide polymorphisms associated with drug resistance in *pfmdr1* and *pfcrt*. Black and striped bars show the frequency of infections carrying alleles associated with reduced sensitivity to lumefantrine i.e., *pfmdr1* N86, 184F, D1246, and *pfcrt* K76. The number of samples genotyped is shown in brackets

PCR success rates for the nested PCRs for determining SNPs in *pfmdr1* N86Y and *pfcrt* K76T were 100% (179/179); PCR success rates for *pfmdr1* Y184F and D1246Y were 96.1% (172/179) and 96.6% (173/179), respectively (Table 2). The *pfmdr1* N86, 184F, D1246 and *pfcrt* K76 alleles that have been associated with reduced sensitivity to lumefantrine were present in 83.8% (150/179), 16.9% (29/172), 100.0% (173/173), and 8.4% (15/179) of all infections, respectively, with similar distribution across the four provinces (Fig. 2). The most common *pfmdr1* haplotype was NYD present in 72.9% (121/166) of the samples, followed by *pfmdr1* YYD in 22.9% (38/166), and *pfmdr1* NFD in 15.1% (25/166) of the samples. The *pfcrt* 76T allele that is associated with chloroquine resistance was found in 98.3% (176/179) of the samples.

**Table 2:** Prevalence of SNPs in *pfk13*, *pfcoronin*, *pfcrt*, and *pfmdr1* in Pakistan, 2018–2019

Gene	N	Wild Type^a^ N (%)	Mutant^b^ N (%)	Mixed^c^ N (%)
*Pfk13*	121	121 (100)	0 (0)	0 (0)
*Pfcoronin*	149	149 (100)	0 (0)	0 (0)
*Pfmdr*1 N86Y	179	137 (76.5)	29 (16.2)	13 (7.3)
*Pfmdr*1 Y184F	172	143 (83.1)	18 (10.5)	11 (6.4)
*Pfmdr*1 D1246Y	173	173 (100)	0 (0)	0 (0)
*Pfcrt* K76T	179	3 (1.7)	164 (91.2)	12 (6.7)

^a^ Wild type alleles include *Pfmdr1* N86, Y184, D1246, and *Pfcrt* K76

^b^ Mutant alleles include *Pfmdr1* 86Y, 184F, 1246Y, and *Pfcrt* 76T

^c^ Mixed alleles include *Pfmdr1* N86Y, Y184F, D1246Y and *Pfcrt* K76T mixed infections

## Discussion

Pakistan has set a target to reduce the malaria burden by 75% in high endemic districts and to eliminate malaria in low endemic districts by 2020 [2]. However, if artemisinin resistance continues to spread westward from neighbouring regions in South East Asia, then efforts in further controlling and eliminating malaria will be largely hampered [38]. Early surveillance of antimalarial drug resistance through molecular epidemiological studies, is in such, essential for achieving these targets. This study provides an update on the current prevalence of molecular markers associated with drug resistance in Pakistan after the introduction of AL as first-line treatment in 2017, including a first baseline assessment of polymorphisms in the *pfcoronin* gene which have been associated with decreased in vitro sensitivity to artemisinin.

No SNPs associated with artemisinin resistance were observed in the *pfk13* propeller domain, nor in *pfcoronin*. One other study has to date, assessed the prevalence of polymorphisms in *pfk13* in Pakistan [39]. This study reported nine non-synonymous and four synonymous mutations among 209 samples collected in 2016–2017. Each SNP was reported in less than 1% of the samples, and none of these SNPs corresponded to mutations that have been associated with artemisinin resistance. These findings are in line with in vivo assessments of ACT efficacy in Pakistan. There has been no evidence of reduced ACT efficacy in Pakistan as of yet, with adequate clinical and parasitological responses of greater than 98% after treatment with AS + SP, AL, and dihydroartemisinin-piperaquine [40]. However, recent increases in AS + SP treatment failures in the North Eastern states of India, bordering with Myanmar, is a matter of concern for the continued westward spread of ACT resistance across from Southeast Asia [17]. Especially since molecular data have indicated that resistance to the SP partner drug is being established in Pakistan, although mutations that confer a high risk of SP treatment failure are still rare or non-existent [39, 40].

AL, on the other hand, remains efficacious in the North Eastern region of India [41, 42], and is currently the recommended first-line treatment for *P. falciparum* in the whole of the WHO Eastern Mediterranean Region, covering Afghanistan, Djibouti, Pakistan, Somalia and Sudan [1]. The efficacy of AL has been monitored in each of these countries, except in Djibouti. All therapeutic efficacy studies in these countries have shown low rates (< 5%) of AL treatment failure, supporting its continued use as first-line treatment. However, whilst no SNPs associated with artemisinin resistance were observed in *pfk13* and *pfcoronin*, the *pfmdr1* N86 and D1246 alleles which have been associated with reduced sensitivity to lumefantrine [25, 28–36] were present in 83.8% and 100% of the isolates in this study, respectively (Table 2). The *pfmdr1* N86 allele in particular, as well as increased *pfmdr1* copy number, have shown to be significant independent risk factors for recrudescence in patients treated with AL [36]. The prevalence of *pfmdr1* N86 has increased since the introduction of ACT in Pakistan in 2007, with a prevalence ranging from 55 to 67% in 2005–2007, and 96% in 2011, (Table 3) [43–45]. The p*fmdr1* D1246 has been consistently reported in 100% of isolates since 2007. *Pfmdr1* copy number was not analysed in this study due to its previously reported very low or non-existent presence in Pakistan.

**Table 3:** Prevalence of polymorphisms in *pfmdr1* and *pfcrt* before and after introduction of AL in Pakistan

	2005–2007 (N = 240) [43]	2007 (N = 28) [44]	2011 (N = 171) [45]	2018–2019 (N = 179) [current study]
*Pfmdr1* N86Y				
N86	43%	67%	80%	77%
86Y	45%	33%^a^	4%	16%
N86Y (mixed)	12%	Not stated^a^	16%	7%
*Pfmdr1* Y184F				
Y184	ND	ND	75%	83%
184F	ND	ND	0%	11%
Y184F (mixed)	ND	ND	25%	6%
*Pfmdr1* D1246Y				
D1246	ND	100%	100%	100%
1246Y	ND	0%	0%	0%
D1246*Y* (mixed)	ND	0%	0%	0%
*Pfmdr1* CNV				
*S*ingle copy	99.6% (231/232)^b^	ND	100%	ND
Multiple copy	0.4% (1/232)^b^	ND	0%	ND
*Pfcrt* K76T				
K76	7%	0%	0%	2%
76T	93%	100%	100%	91%
K76T (mixed)	0%	0%	0%	7%

^a^ Proportion of infections containing *pfmdr1* 86Y, proportion of which were mixed infections not stated

^b^ (n/number of samples successfully analysed)

ND, not determined; CNV, copy number variation

Over a decade of wide-scale use of AL, mainly in African counties, has selected for *pfcrt* K76 and the *pfmdr1* haplotype N86/184F/D1246, with a parallel decline in *pfcrt* 76T and the *pfmdr1* 86Y/Y184/1246Y haplotype [31, 33, 35, 36, 46–48]. Parasites harbouring the *pfmdr1* NFD haplotype have shown to able to re-infect patients whose lumefantrine blood concentrations were 15-fold higher than for parasites carrying the *pfmdr1* YYY haplotype [32]. Despite this wide scale selection of SNPs associated with reduced sensitivity of lumefantrine, AL remains highly effective across sub-Saharan Africa [49]. However, tracking changes in the prevalence of molecular markers of drug resistance can be a sensitive indicator of the selection of parasite populations, and may signal early decreases in drug susceptibility. In addition, decreasing efficacy of the partner drug may expose the artemisinin component of the ACT to selective pressure, and could facilitate emergence of new foci of resistance to artemisinin, as observed in the Greater Mekong Subregion [36]. Hence, in addition to therapeutic efficacy studies, further monitoring of the selection of molecular markers associated with both artemether and lumefantrine resistance in Pakistan, as in this study, is considered to be a critical tool to detect and prevent the spread of artemisinin resistance, and for preserving the efficacy of AL in the area [36].

Finally, the observed high prevalence (98.3%) of the chloroquine resistance associated *pfcrt* 76T allele is in line with previous assessments [43–45, 50, 51], and provides molecular evidence of chloroquine resistant *P. falciparum* in Pakistan. Although chloroquine was removed from the national guidelines for treatment of *P. falciparum* malaria more than 10 years ago, chloroquine is still recommended in the treatment of *P. vivax* and unconfirmed malaria infections [1]. Improper diagnosis of malaria, due to presumptive diagnosis based on clinical grounds and/or lack of diagnostic facilities (which are common practices in resource-limited countries such as Pakistan), is likely to sustain a high exposure of *P. falciparum* to chloroquine, maintaining the near fixation of the chloroquine resistant *pfcrt* 76T allele.

## Conclusions

This study provides an update on the current prevalence of molecular markers associated with reduced *P. falciparum* sensitivity to artemether and/or lumefantrine in Pakistan. No mutations associated with artemisinin resistance were observed in *pfk13* or *pfcoronin*. However, the prevalence of the *pfmdr1* N86 and D1246 alleles, that have been associated with decreased susceptibility to lumefantrine, remain high. Although clinical and molecular data suggest that the current malaria treatment guidelines for *P. falciparum* are presently effective, close monitoring for artemisinin and lumefantrine resistance will be critical to ensure early detection and enhanced containment of emerging ACT resistance spreading across from Southeast Asia.

## Data Availability

The datasets used and/or analysed during the current study are available from the corresponding author on reasonable request.

## Abbreviations

ACT: Artemisinin-based combination therapy
AL: Artemether–lumefantrine
AS + SP: Artesunate-sulfadoxine-pyrimethamine
DI: Dera Ismail
DNA: Deoxyribonucleic acid
FR: Frontier region
KPK: Khyber Pakhtunkhwa
PCR: Polymerase chain reaction
*Pfcoronin*: *Plasmodium falciparum* coronin gene
*Pfcrt*: *Plasmodium falciparum* chloroquine resistance transporter gene
*Pfk13*: *Plasmodium falciparum* Kelch 13 gene
*Pfmdr1*: *Plasmodium falciparum* multidrug resistance 1 gene
RFLP: Restriction fragment length polymorphism
SNP: Single nucleotide polymorphism
WHO: World Health Organization
